# Efficacy of Sorafenib Combined With Immunotherapy Following Transarterial Chemoembolization for Advanced Hepatocellular Carcinoma: A Propensity Score Analysis

**DOI:** 10.3389/fonc.2022.807102

**Published:** 2022-04-08

**Authors:** Jian Qin, Yusheng Huang, Hanjing Zhou, Shouhui Yi

**Affiliations:** ^1^Department of Oncology, the Second Affiliated Hospital of Chongqing Medical University, Chongqing, China; ^2^Department of Oncology, the First Affiliated Hospital of Chongqing Medical University, Chongqing, China

**Keywords:** HCC, TACE, ICIs, immunotherapy, multi-target drugs

## Abstract

**Aim:**

The aim of the study is to compare the efficacy and safety of monotherapy with a sequential immune checkpoint inhibitor (ICI) programmed cell death protein-1 (PD-1) and its combination with multi-target drug sorafenib after transcatheter arterial chemoembolization (TACE) for advanced hepatocellular carcinoma (HCC).

**Methods:**

We conducted a retrospective evaluation of patients with advanced HCC who had received sequential PD-1 sorafenib (duplex group, n = 25) or monotherapy PD-1 alone (PD-1 group, n = 41) after TACE during April 2018–September 2021. Propensity score matching (PSM) was applied to correct the selection bias, and 22 pairs were created. The objective response rate (ORR), duration of the overall response (DOR), disease control rate (DCR), progression-free survival (PFS), overall survival (OS), and adverse events were analyzed for both groups.

**Results:**

After PSM, the median PFS (7.63 vs. 2.9 months; p = 0.0335) was significantly longer for the duplex group than for the PD-1 group. The median OS (21.63 vs. 16.43 months; p = 0.103) was longer for the duplex group than for the PD-1 group, albeit without any statistical difference. The CR rate, ORR, DCR, and PFS rates at the first, third, and sixth months were higher for the duplex group than for the PD-1 group, wherein the PFS rate of the third and sixth months were statistically different. The OS rates at the sixth, 12th, and 18th months were better for the duplex group than for the PD-1 group, while the 18th-month OS rate (54.5% vs. 33.9%, p = 0.030) were statistically different between them. The most common adverse events after TACE included liver function injury, leukocytopenia, and thrombocytopenia, albeit without any statistical differences between the groups. Cox regression analysis showed that sorafenib combined immunotherapy after TACE and the achieving of CR or PR during the treatment were independent factors affecting PFS. Moreover, CNLC stage-IIIa, TACE frequency ≤2, and achievement of CR or PR were independent influencing factors of OS.

**Conclusions:**

Sequential PD-1 combined with sorafenib therapy after TACE for advanced HCC treatment is safe and effective, especially for patients with good initial treatment response, to further improve the disease prognosis.

## 1 Introduction

Hepatocellular carcinoma (HCC) is one of the diseases contributing a high morbidity and mortality rates in the world, with 393,000 new cases of liver cancer and 369,000 liver cancer-related deaths reported every year in China alone, which accounts for >50% of all liver cancer cases worldwide according to the *WHO Global Cancer Statistics Report* in 2018 (1). Early-stage HCC can be surgically treated. However, HCC is usually latent in onset, rapid in progression, easy in recurrence, and poor in prognosis. Therefore, most patients with HCC are often diagnosed at an advanced stage, thereby missing the opportunity for timely surgical intervention. Transcatheter arterial chemoembolization (TACE) is a common treatment adopted for intermediate or advanced HCC (2). However, as a heterogeneous disease, there are large differences in the liver functions and tumor loads and also in the effects of TACE treatment across advanced HCC patients. Especially, for intermediate-stage patients with tumors exceeding UP-to-7 criteria, TACE not only has poor efficacy but may also damage the liver reserve, thereby leading to poor prognosis. For advanced HCC, TACE alone is therefore insufficient to achieve satisfactory efficacy owing to issues such as vascular invasion and distant metastasis, which, therefore, warrants the need for the establishment of combination therapy.

Immunotherapy for unresectable HCC is currently a hot research topic. The immune system is insensitive to foreign body reactions in the liver owing to its functional requirement. In this regard, it is easier for tumor cells in the liver to escape the surveillance and subsequent attack of the immune system, also called “immune evasion”. Immuno-checkpoint inhibitors (ICIs), such as PD-1/PD-L1, enable autoimmune cells to contribute to the anti-tumor effect by eliminating immunosuppression. On the other hand, although the tumor tissue hypoxia is aggravated after TACE, which induces necrosis and apoptosis of the tumor cells, tumor angiogenesis also gets promoted, thereby indirectly leading to the recurrence and metastasis of HCC patients after TACE. As common multi-target drugs (MTDs) for intermediate or advanced HCC, sorafenib and lenvatinib target *VEGFR*-1/2/3 and block neovascularization required for tumor growth. When targeting anti-angiogenesis, MTDs can simultaneously target multiple proteins that promote tumor growth such as *RET*, *FLT3*, and *BRAF*, which contribute to the overall anti-tumor effect. Therefore, the combination therapy of TACE with MTDs can suppress tumor angiogenesis while also killing the tumor cells, thus contributing to better therapeutic effectiveness for anti-tumor treatment. It has been confirmed through the TACTICS study (3), presented at the 2018 ASCO meeting, that TACE combined with sorafenib therapy led to a median PFS advantage of 11.7 months (25.2 months versus 13.5 months, P <0.01) for early or intermediate stage patients with unresectable HCC without any vascular invasion. However, for stage-III HCC patients complicated with large masses, tumor vascular invasion, or even distant metastasis, the sequential therapeutic regimen after TACE remains inconclusive.

Theoretically, after TACE, tumor cells undergo necrosis and disintegration owing to hypoxia and damage from chemotherapy drugs, thereby releasing a large number of tumor antigens. Moreover, it can cause local inflammation, promote lymphocyte infiltration, and activate the immune system. By removing immunosuppression, PD1/PD-L1 enables immune cells to further contribute to the overall anticancer effect. Meanwhile, MTDs can induce the normalization of tumor vessels while antagonizing hypoxia-induced angiogenesis after TACE. Furthermore, they can improve the microenvironment for tumor tissues to provide a favorable microenvironment for immunotherapy. In this study, we thus investigated the efficacy and safety of PD-1 therapy combining sorafenib after TACE and preliminarily explore the predictive factors affecting the efficacy of patients.

## 2 Objects and Methods

### 2.1 Objects

#### 2.1.1 Patients

Patients diagnosed with advanced HCC and treated with TACE sequential PD-1 immunotherapy in the Second Affiliated Hospital of Chongqing Medical University from April 2018 to September 2021 were enrolled. The subjects were categorized into the duplex and PD-1 groups based on their treatment course.

#### 2.1.2 Inclusion Criteria

(1) Patients who were clinically or pathologically diagnosed with HCC according to the diagnostic criteria of China’s Code for The Diagnosis and Treatment of Primary Liver Cancer (2017 Edition); (2) Clinical stage: China Liver Cancer stage IIIA or IIIB or Barcelona Clinic liver cancer stage C; (3) Patients who underwent sequential PD-1 immunotherapy after TACE as the initial treatment, with an interval between TACE and PD-1 treatment of <2 months; (4) KPS score >90; (5) A to B classification on the Child–Pugh liver function scale; and (6) Patients with complete follow-up data available.

#### 2.1.3 Exclusion Criteria

(1) Patients who underwent sequential PD-1 therapy for >2 months after TACE; (2) Patients suffering from other accompanying primary tumors; and (3) Patients suffering from other accompanying serious primary diseases.

### 2.2 Methods

#### 2.2.1 Treatment

TACE was intubated through the femoral artery by the Seldinger method, and a super-selective catheter was inserted into the tumor supplying artery. Drugs and dosage: 20–80 mg pirarubicin/epirubicin, 10–50 mg loplatin, 3–6 mg raltetrexed, and 6–20 ml iodide oil. After performing TACE, the PD-1 group was treated with sequential PD-1 (sintilimab or camrelizumab; 200 mg every 21 days), while the duplex group received the combination therapy of sequential PD-1 (sintilimab or camrelizumab; 200 mg every 21 days) and sorafenib (400 mg twice daily) on day 4 after the initial TACE. Then, sorafenib was administered on an interrupted schedule, at a 4–7-day interval before and after each subsequent TACE. In case of clinically significant toxicity of grade 2 (according to the National Cancer Institute Common Terminology Criteria for Adverse Events, version 4.0), the sorafenib dose was reduced, delayed, or briefly interrupted. Dose escalation or sorafenib rechallenge was determined when the toxicity was diminished and the patient could well tolerate the treatment. After the disease progression, the patients received a systemic treatment (Regorafenib) or symptomatic supportive therapy.

#### 2.2.2 Observing Indexes

Enhanced computed tomography (CT) or magnetic resonance imaging (MRI) was performed once every 1–2 months initially, and then once every 2 months after 6 months. All the patients were evaluated according to the mRECIST criteria and classified as follows: complete response (CR): CT or MRI showed no enhancement in the arterial phase of all target lesions; partial response (PR): a 30% reduction in the total diameter of the target lesions (arterial enhancement); stable disease (SD): the total diameter reduction of target lesions (arterial enhancement) did not reach PR or the increase did not reach PD; disease progression: (PD) target lesions (arterial enhancement) showed a 20% increase in the total diameter or new lesions were detected. Survival analysis: overall survival (OS) time was defined as the time from initial treatment with TACE to death or the end of follow-up; progression-free survival (PFS) was defined as the time from the initial treatment with TACE to the first occurrence of tumor progression or death or the end of follow-up; the optimal duration of response (DOR) was defined as the time from CR or PR to initial PD or death or the end of follow-up. Major complications (grade 3 or 4 toxicity) were assessed and compared with reference to the specifications of the National Cancer Institute Common Terminology Criteria for Adverse Events (version 4.0).

#### 2.2.3 Statistical Methods

Propensity score matching (PSM) was performed to minimize the effects of selection bias and potential confounders. The independent variables entered into the propensity model included age, sex, maximal tumor size, tumor type (nodular/lumpy/massive/diffuse), presence of portal vein cancer thrombus invasion, the levels of alpha-fetoprotein (AFP), and the achievement of CR or PR during treatment. The model was then applied to provide one-to-one matches between the two groups using the nearest-neighbor method. These groups were compared before PSM using the Student’s *t*-test for continuous variables and the Chi-squared test for categorical variables. After PSM, the study groups were compared by paired *t*-tests for continuous variables and the McNemar test or marginal homogeneity test for categorical variables.

All analyses were performed by using the Statistical Package for the Social Science (SPSS) version 26. Measurement data were evaluated by T-Test. Counting data were assessed by χ^2^. As for survival analysis, univariate analysis was performed by the Kaplan–Meier method, and then evaluated by Log-rank test, while multivariate analysis was conducted by using the Cox Regression Model. *p <*0.05 was considered to indicate statistical significance. PFS and OS rates were calculated using the Kaplan–Meier method and paired Prentice–Wilcoxon test after PSM. Variables showing associations with a p <0.05 and with a marginal significance in the univariable analysis (p <0.1) were entered into the multivariable model. After PSM, stratified Cox regression analysis and doubly robust estimation were adjusted for factors significant in the univariable analysis to identify the independent predictive function of the treatment type with regard to the OS.

## 3 Results

### 3.1 Baseline Characteristics

A total of 66 patients who had received PD-1-sorafenib (n = 25) or PD-1 monotherapy (n = 41) after TACE as the initial treatment for advanced HCC were screened. The baseline characteristics of the patients are summarized in **Table 1**. Before PSM, the PD-1 group showed a younger mean age (48.66 ± 11.79 vs. 55.92 ± 10.84, respectively; p = 0.015) and a lower probability of intrahepatic metastasis (21.9% vs. 52%; p = 0.012) when compared with the duplex group. The proportion of patients with lower AFP levels in the duplex group was higher than that in the PD-1 group (48% vs. 19.5%; p = 0.015). No significant difference was observed in terms of sex, CNLC stage, Child–Pugh score, tumor type, hepatitis B antigen expression, HBV-DNA index, portal vein cancer thrombus, extrahepatic metastasis, the number of patients who achieved CR/PR, and the times of TACE between the two study groups (*p >*0.05). All 66 patients were treated with sequential PD1 mab after TACE, namely, carrellizumab (n = 42), sintilimab (n = 24), and the median time of ICIs treatment was 4.9 months (n = 7). After PSM, the baseline characteristics of the patients were noted to be more balanced than those before treatment, with no statistically significant differences observed in terms of age, intrahepatic metastasis, and the AFP levels between the two study groups.

**Table 1:** Baseline characteristics of all study patients.

Characteristic	Before Matching			After Matching		
	Duplex Group	PD-1 Group	*P*-value	Duplex Group	PD-1 Group	*P*-value
	(n = 25)	(n = 41)		(n = 22)	(n = 22)	
Age (x ± s, years)	55.92 ± 10.84	48.66 ± 11.79	0.015	53.82 ± 9.77	53.41 ± 10.01	0.892
Gender (female/male)	2/23	5/36	0.901	1/21	1/21	1.000
CNLC staging (IIIa/IIIb)	20/5	28/13	0.300	17/5	16/6	0.728
Child–Pugh (A/B)	24/1	38/3	1.000	21/1	19/3	0.600
Gross type						
nodular	6	3	0.122	5	2	0.410
lumpy	8	14	0.858	7	11	0.220
massive	10	23	0.205	9	8	0.757
diffuse	1	1	1.000	1	1	1.000
Massive (No/Yes)	15/10	18/23	0.205	13/9	14/8	0.757
Intrahepatic metastasis (No/Yes)	13/12	9/32	0.012	11/11	8/14	0.361
Hepatitis B antigen expression						
negative	5	3	0.269	3	3	1.000
small positive	14	30	0.151	13	15	0.531
large positive	6	8	0.665	6	4	0.472
HBV-DNA index (≥1 × 10^3^/<1 × 10^3^)	9/16	21/20	0.228	9/13	13/9	0.228
Portal vein cancer thrombus (No/Yes)	5/20	10/31	0.680	4/18	5/17	1.000
Extrahepatic metastasis (No/Yes)	20/5	28/13	0.300	17/5	16/6	0.728
AFP						
<20 ng/L	12	8	0.015	10	4	0.052
20–399 ng/L	7	15	0.473	7	10	0.353
≥400 ng/L	6	18	0.103	5	8	0.322
Optimal response to CR/PR (No/Yes)	9/16	24/17	0.076	9/13	10/12	0.761
PD-1/PD-L1	25/0	41/0		22/0	22/0	
TACE times (>2 times/≤2 times)	11/14	13/28	0.314	9/13	7/15	0.531

Before matching, statistically significant differences were noted between the two study groups in terms of age, whether intrahepatic metastases were combined, and the methemoglobin levels (p < 0.05). After matching, no statistical differences were noted between the two study groups in terms of age, sex, CNLC staging, Child–Pugh classification, tumor gross classification, intrahepatic/extracellular metastasis, portal vein tumor thrombus, hepatitis-B surface antigen expression, HBV-DNA index, AFP, the number of cases achieving CR/PR, and TACE times (p > 0.05).

### 3.2 Effectiveness and Safety

**Figure 1:**
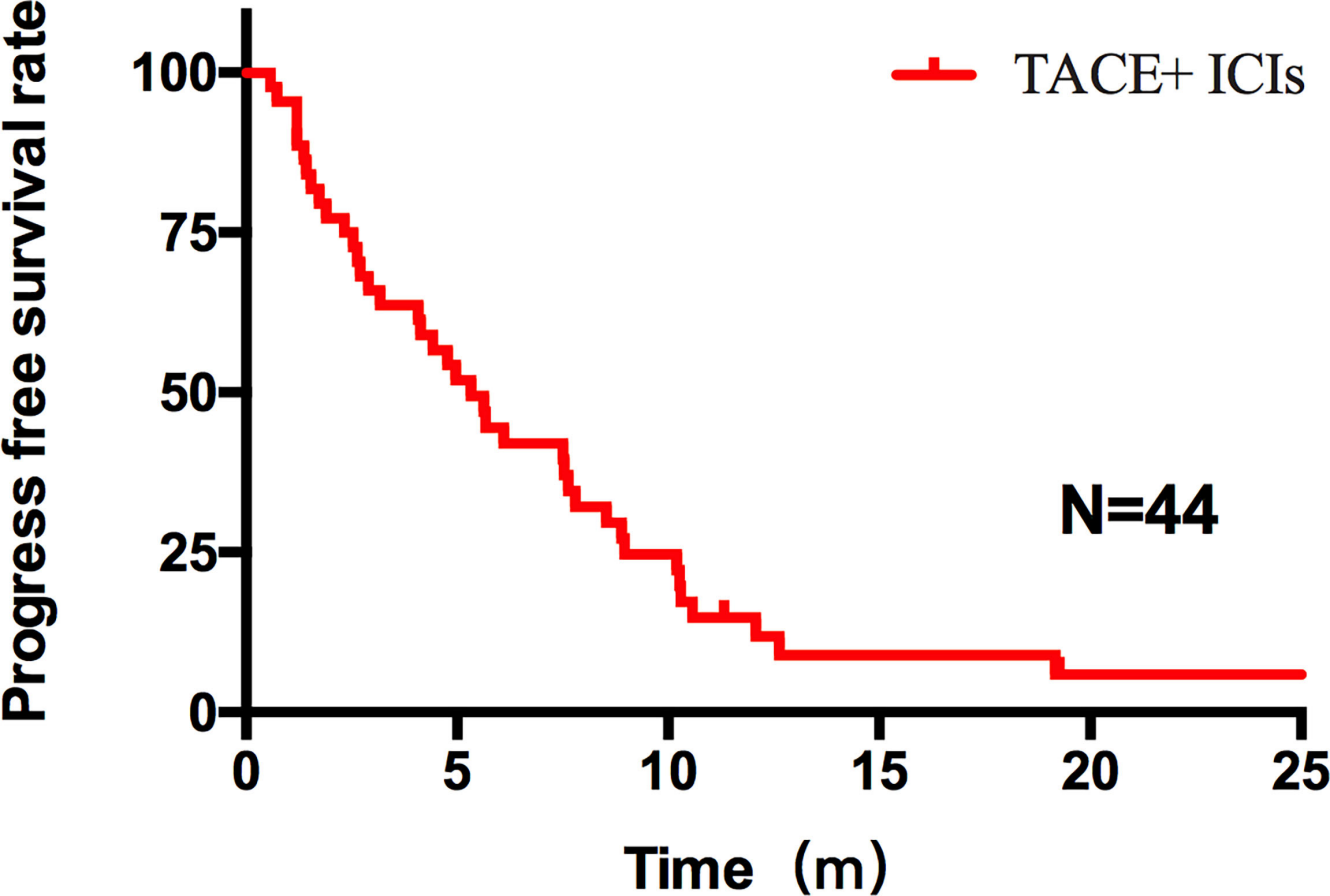
PFS of all patients after TACE–ICIs combined therapy. Patients after TACE-ICIs combined therapy achieved a median progression-free survival of 5.33 months.

**Figure 2:**
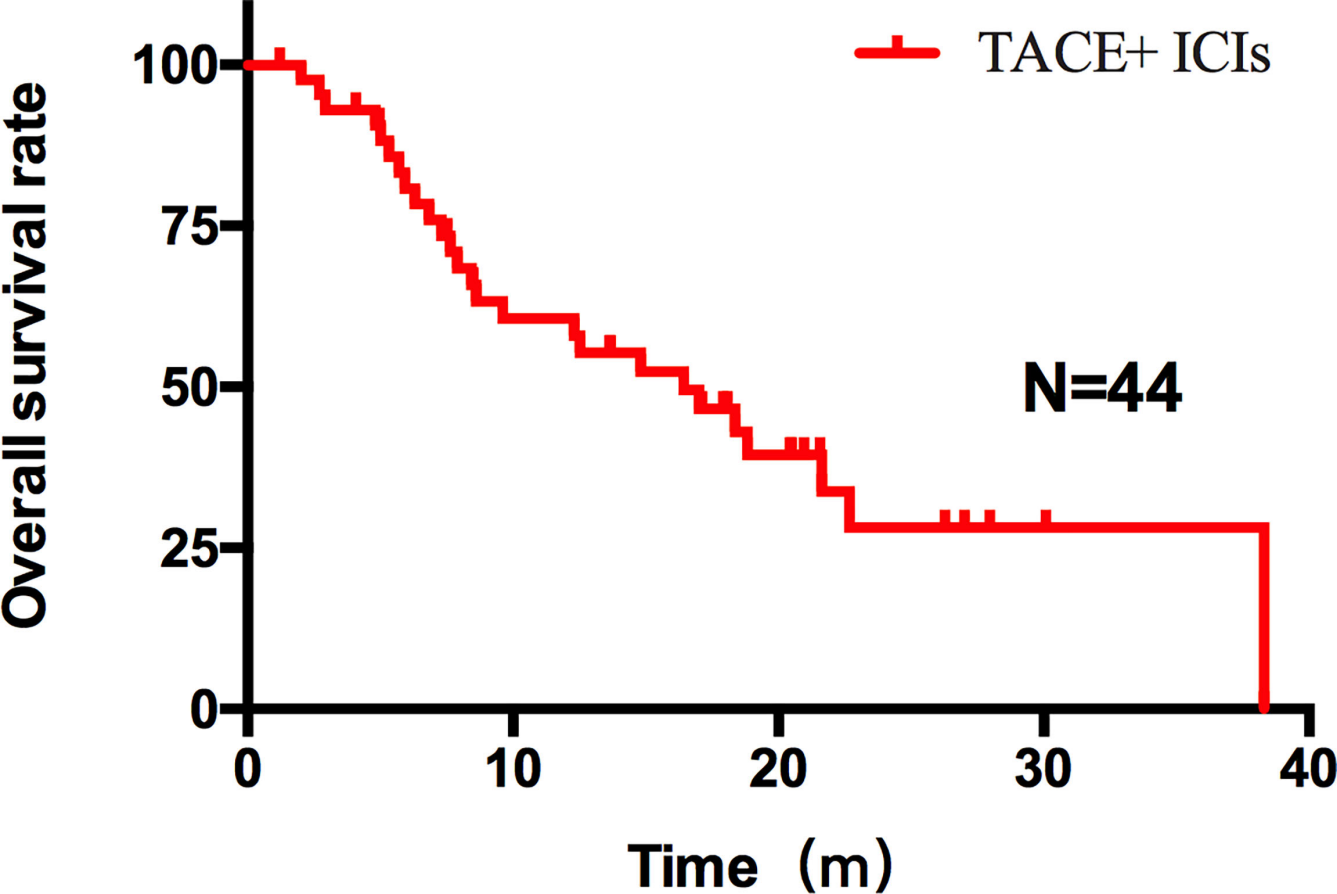
OS of all patients after TACE–ICIs combined therapy. Patients after TACE-ICIs combined therapy achieved a median OS of 16.43 months.

**Figure 3:**
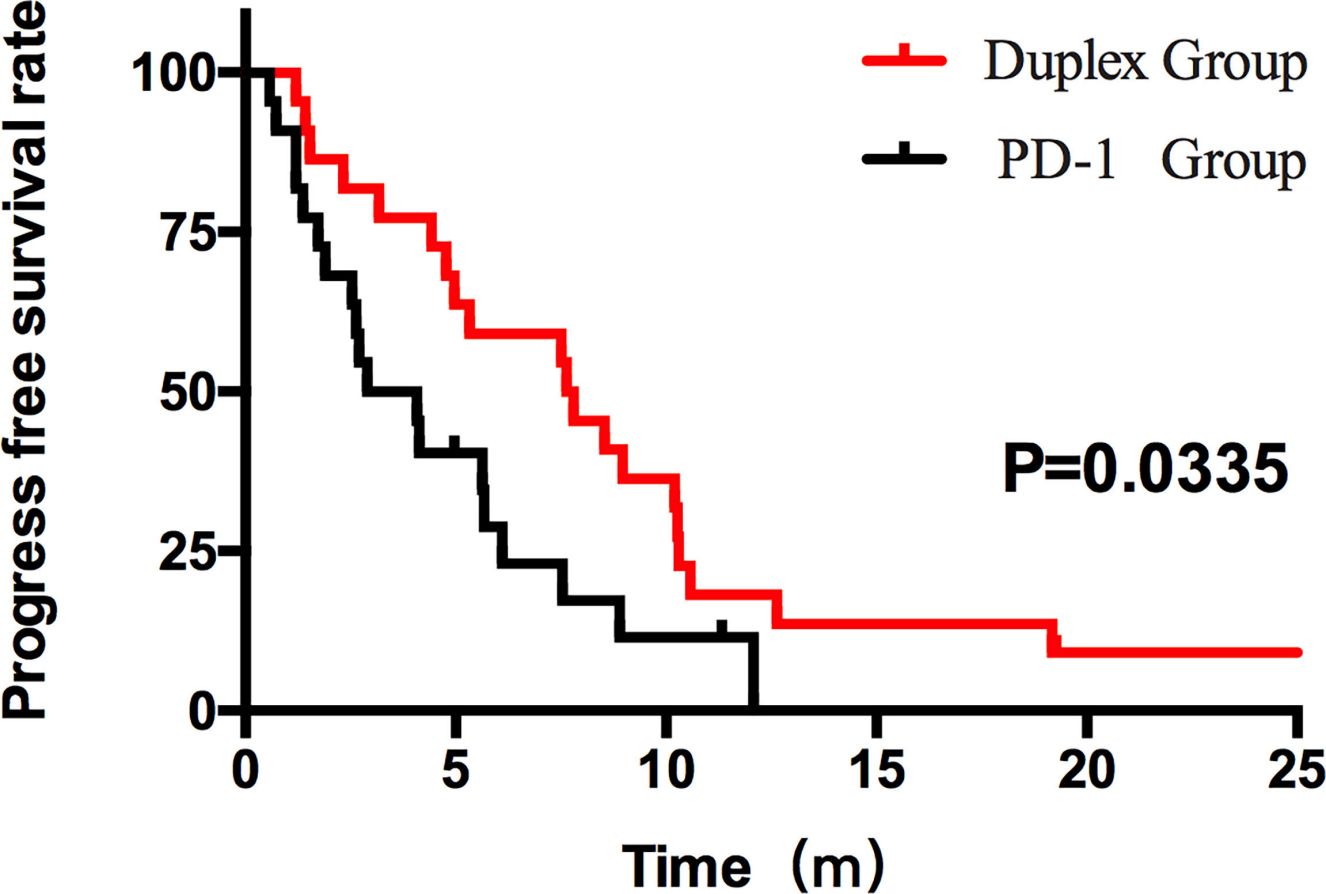
PFS of the duplex group and the PD-1 group. The PFS results between the two groups were statistically significant (7.63 months vs. 2.90 months, p = 0.0335, HR 0.44, 95% CI [0.236, 0.942]).

**Figure 4:**
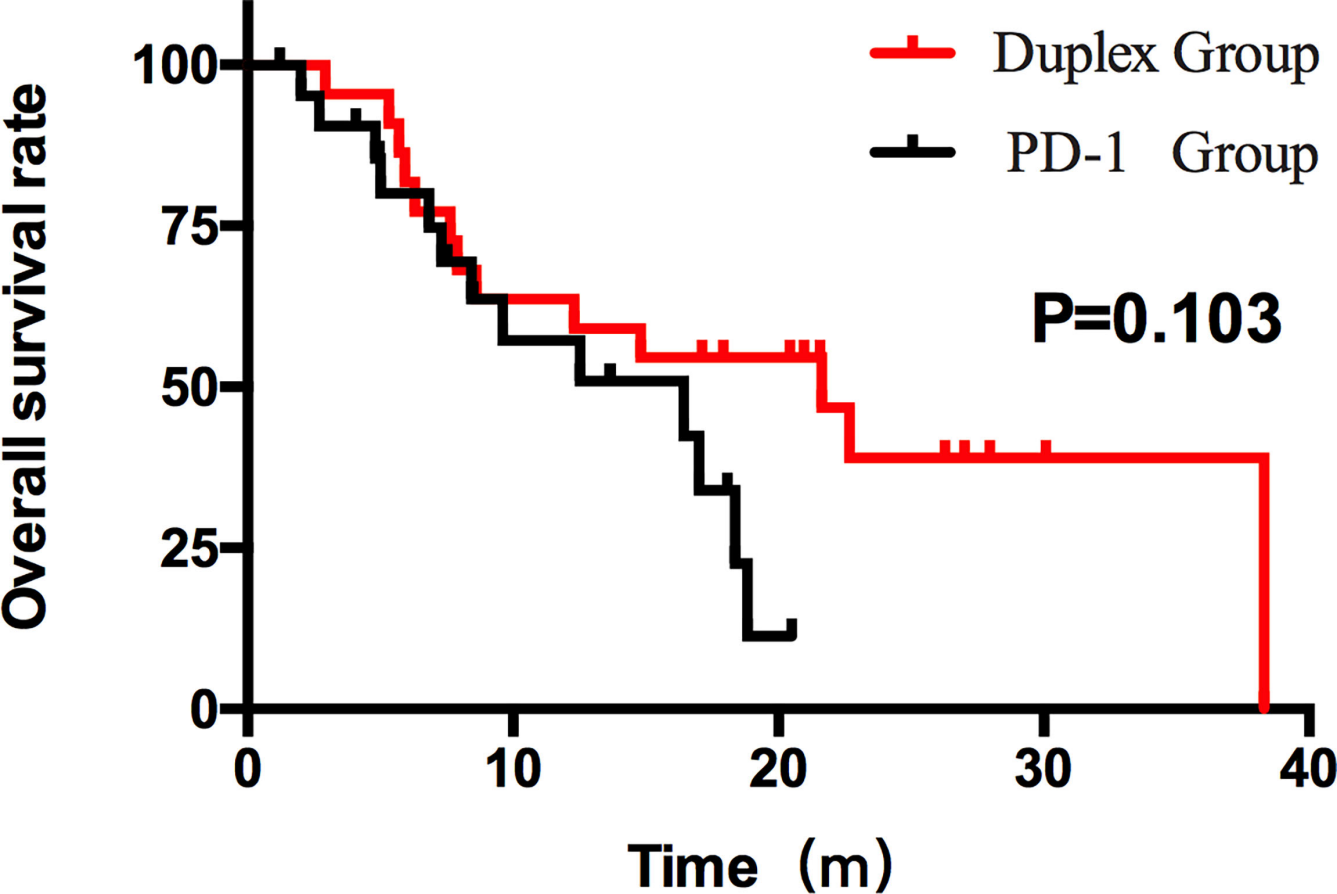
OS of the duplex group and the PD-1 group. The OS results between the two groups were different (21.63 months and 16.43 months). However, the difference was not statistically significant (*p > *0.05).

Patients who combined the sequential PD-1 immunotherapy after TACE as the initial treatment achieved a median PFS of 5.33 months (**Figure 1**) and a median OS of 16.43 months (**Figure 2**). The median follow-up time was 8.5 months. Meanwhile, their object response rate, median DOR, and the disease control rate were 56.8%, 7 months, and 84.09%, respectively. The median PFS of the duplex group was 7.63 months when compared with 2.9 months for the PD-1 group, and the difference was statistically significant (*p* = 0.033) (**Figure 3**), HR 0.44, and 95% CI [0.236, 0.942]. The PFS rates at the first, third, and sixth months of the duplex group were all higher than those of the PD-1 group (100%, 81.8%, and 59.1% vs. 90.9%, 50.0%, and 28.9%), in which the PFS rates at the third (*p* = 0.026) and sixth (*p* = 0.014) months were statistically different. Among the 66 patients, 3 patients were recently lost to follow-up, and the OS data in the two groups showed a maturity of 59.09%. The median OS of the duplex group was better than that of the PD-1 group, albeit the difference was not statistically significant (OS: 21.63 months vs. 16.43 months, *p* = 0.103) (**Figure 4**). The OS rates at the sixth, 12th, and 18th months of the duplex group were better than those of the PD-1 group, wherein the OS rates at the 18th month (54.5% vs. 33.9%, p = 0.030) were statistically different. As for the duplex group, 9 patients achieved CR, 4 achieved PR, 8 were at the SD state, and 1 had PD. However, in the PD-1 group, 4 patients achieved CR, 8 achieved PR, 4 were at the SD state, and 6 had PD. The CR rate, ORR, and DCR of the duplex group were all higher than those of the PD-1 group, albeit without a statistically significant difference (*p >*0.05) (**Table 2**).

**Table 2:** Survival analysis and therapeutic effectiveness between the two study groups.

Index	Duplex Group (n = 22)	PD-1 Group (n = 22)	*p*-value
PFS (month) (95% CI)	7.63	2.90	0.033
	(3.95–11.30)	(1.22–4.57)	
PFS rate (%)			
1 month	100.0	90.9	0.488
3 months	81.8	50.0	0.026
6 months	59.1	28.9	0.014
OS (month) (95% CI)	21.63 (9.90–33.35)	16.43 (5.04–27.81)	0.103
OS rate (%)			
6 months	81.8	80.1	1.000
12 months	63.6	57.3	0.242
18 months	54.5	33.9	0.030
Efficacy (cases)			
CR	9	4	0.099
PR	4	8	
SD	8	4	
PD	1	6	
ORR (%)	59.09	50.00	0.761
DCR (%)	95.45	72.72	0.095

The duplex group achieved a better median PFS, OS compared with the PD-1 group, and the difference in the median PFS was statistically significant (p = 0.033). In terms of the PFS rate, OS rate at the first, third, and sixth months, the CR rate, ORR, and DCR rate, the duplex group showed a better outcome than the PD-1 group, and the OS rate at the 18th month, the PFS rate at the third and sixth months were statistically significant (p < 0.05) between the two study groups.

In terms of safety, no treatment-related death was noted in the two groups, and the main adverse effects after TACE included liver function injury, leukocytopenia, and thrombocytopenia, which could be recovered in a short time after the expectant treatment. No statistical significance was recorded for grades >3–4 adverse reactions between the two groups. Post-embolization syndrome (such as fever, pain, loss of appetite, nausea, and vomiting) was recorded in 16 patients of the duplex group when compared with that in 17 patients of the PD-1 group, indicating no statistical difference. Grade 1–2 thyroid dysfunction was recorded in all 16 patients. No cardiac dysfunction patient showed any significant increase in cTn, change in ECG, or complication with clinical symptoms. As unique adverse reactions of patients in the duplex group, grade 1–2 hand–foot–skin reactions were noted in 7 patients, namely, pain, blisters, and peeling, which could be recovered after drug withdrawal, dosage reduction, and symptomatic treatment (**Table 3**).

**Table 3:** Safety assessment of the two study groups.

Toxic effects	Total (n = 44)	Duplex Group (n = 22)	PD-1 Group (n = 22)	*p*-value
Leukopenia	21	9	12	0.365
Grade 1–2/case	18	8	10	
Grade 3–4/case	3	1	2	1.000
Thrombocytopenia	29	14	15	0.750
Grade 1–2/case	18	10	8	
Grade 3–4/case	11	4	7	0.316
Hepatocyte dysfunction	44	22	22	1.000
Grade 1–2/case	34	18	16	
Grade 3–4/case	10	4	6	0.472
Thyroid dysfunction	16	7	9	0.531
Cardiotoxicity	0	0	0	1.000
Hand–foot–skin reaction	7	7	0	0.009
Post-embolization syndrome	33	16	17	0.728
Nausea	17	10	7	0.353
Vomiting	10	6	2	0.240
Pain	13	8	5	0.322
Fever	21	11	10	0.763

No significant difference was recorded in adverse events, such as liver function injury, leukopenia, thrombocytopenia, thyroid dysfunction, cardiac dysfunction, and post-embolization syndrome between the two groups after TACE (p >0.05), and the hand–foot–skin reactions were unique to the duplex group (p < 0.05).

### 3.3 Factors Affecting PFS and OS

After PSM, univariate analysis was performed on age, CNLC staging, large mass type (maximum diameter ≥10 cm), intrahepatic metastasis, HBV-DNA index (>1 × 10^3^/<1 × 10^3^), AFP expression before TACE (<20/20–399/≥400 ng/L), achieving CR or PR, and also the ICIs-MTDs combination therapy after performing TACE and TACE frequency ≤2, among other factors. These results demonstrate that massive liver cancer (*p* = 0.041), achieving CR or PR during treatment (*p* = 0.000), and conducting combination therapy with MTDs (*p* = 0.037) were the factors affecting PFS. Cox regression analysis was conducted for the abovementioned factors with a significant difference between the two groups, according to the input method. Five factors were screened based on *p <*0.1, namely, massive HCC (*p* = 0.041), achieving CR or PR (*p* = 0.000), and combination therapy with MTDs (*p* = 0.037). The Cox regression and doubly robust estimation adjusted for factors significant in the univariable analysis revealed that the sorafenib combined after TACE (regression coefficient -1.110, Wald value 8.981, *p* = 0.003, HR 0.33, 95% CI [0.159, 0.681]) and achieving CR or PR during treatment (regression coefficient −1.662, Wald value 19.932, *p* = 0.000, HR 0.19, 95% CI [0.091, 0.394]) were independent factors affecting PFS, based on the forward LR method.

The same method was used to perform univariate analysis for OS. The results illustrated that CNLC staging (*p* = 0.022), achieving CR or PR during treatment (*p* = 0.000), and TACE frequency ≤2 (*p* = 0.002) were the factors affecting OS. Variables demonstrating associations with *p <*0.05 and with a marginal significance in the univariable analysis (*p <*0.1) were entered into the multivariable model. Five factors were screened out, namely, massive HCC (*p* = 0.081), achieving CR or PR (*p* = 0.000), combination therapy with MTDs (*p* = 0.090), CNLC staging (*p* = 0.022), and the number of TACE ≤2 (*p* = 0.002). Cox regression analysis revealed that CNLC stage-IIIa (regression coefficient −1.215, Wald value 6.23, *p* = 0.013, HR 0.33, 95% CI [0.114, 0.770]), achieving CR or PR (regression coefficient −0.951, Wald value 4.199, *p* = 0.040, HR 0.38, 95% CI [0.156, 0.959]), TACE frequency ≤2 (regression coefficient −1.498, Wald value 6.821, *p* = 0.009, HR 4.47, 95% CI [1.453, 13.768]) were independent factors affecting the OS of patients.

## 4 Discussion

As per the standard treatment for advanced HCC, the efficacy of TACE was subject to the tumor blood supply, lesion location, and tumor size. Therefore, it is difficult to achieve complete tumor necrosis by TACE alone, and the treated tumor is often prone to recurrence and metastasis. According to the 2020 EDITION of *CSCO Guidelines for the Diagnosis and Treatment of Primary Liver Cancer*, it is preferable to combine TACE with local therapy (such as ablation, surgery, or radiotherapy) and systemic therapy (such as molecular targeted drugs, immunotherapy, or antiviral therapy) to further improve the efficacy of TACE.

With the publishing of CheckMate 040, KEYnote-240, Keynote-224, and SHR-1210 (4–7), PD-1 inhibitors have been included in multiple guidelines at home and abroad and recommended as the second-line therapy for advanced HCC. For the first-line treatment for advanced HCC, although Checkmate 459 failed to achieve its endpoint, it showed better tolerability, higher response rates, and clinically significant OS improvement when compared with sorafenib. Although a single-agent therapy by ICIs showed a low ORR, its combination with TACE has a solid theoretical basis.

First of all, the liver is an immune preferential organ, and HCC is a typical inflammatory malignant tumor (8), which is an ideal place for ICIs to exhibit a role. Strengthening the recognition between immune cells and tumor cells has been recognized as the key to treatment. Furthermore, past studies have indicated a significant increase in the proportion of cytolytic T-lymphocyte after TACE, indicating an immunological enhancement in the body. It is therefore theoretically feasible to conduct ICIs treatment on the basis of inflammatory response and enhanced immunity (9). On the other hand, tumor antigen release is the initiating link among the 7 key links of immunotherapy, such as tumor antigen release, antigen presentation, sensitization, activation, and T-cell migration. Therefore, in combined immunotherapy, increasing tumor neoantigen is bound to enhance the anti-tumor immune response. In addition, a recent study revealed a significant increase in the PD-L1 expression of tumor cells in the local lesions and the PD1/PD-L1 expression of inflammatory cells in an immune microenvironment of HCC patients after TACE (10).

This study focuses on the CNLC stage IIIA and IIIB or BCLC stage C HCC patients who demonstrated PS score 0–1, Child–Pugh grade A–B, and tumor invasion of the blood vessels or distant metastasis. For such patients, TACE can only control the local lesions but does not affect distant metastatic lesions, thereby warranting a combination therapy. For advanced HCC patients after TACE, the tumor burden is significantly reduced in a short time, and the immune status of the patient is also changed. It has been reported that TACE + ICIs + MTDs not only activate cellular immunity but also activate humoral immunity (11). Based on this report, combined therapy can be considered to be theoretically feasible. So far, there have been no published prospective studies on PD-1 monotherapy conducted in combination with TACE or PD-1 with MTDs in combination with TACE. However, two therapeutic modes already exist in clinical practice. In this study, patients with advanced HCC after TACE exhibited a significant reduction in their tumor load in a short time. On the basis of this result, the treatment combined with ICIs achieved a median PFS of 5.33 months, an ORR of 56.8%, and a disease control rate of 84.09%. This result illustrates the advantages of the TACE-ICIs combination therapy for HCC. For stage-IIIb HCC, TACE is recommended as level-II treatment as per the CSCO guidelines. We could confirm through this study that TACE-ICIs combination therapy can achieve good therapeutic effectiveness when a better level-I recommendation is unavailable. In 2016, a small single-arm study (12) using tremelimumab in combination with TACE or RFA for patients with advanced HCC illustrated a median PFS of 7.4 months and a median OS of 13.6 months in 11 patients who received TACE in combination with immunotherapy. Presently, considering the numbers of the Clinical Trial registration, there is a large number of ongoing prospective clinical studies, albeit no more relevant reports are available. Recently, the enrollment of the randomized Phase 3 LEAP-012 Study (ClinicalTrials.gov NCT04246177) has begun. The benefit of adding lenvatinib plus pembrolizumab to TACE in patients with intermediate-stage HCC will be evaluated (13).

As the key to tumor angiogenesis, vascular endothelial growth factor (VEGF) is directly involved in the growth, metastasis, and diffusion of HCC. After TACE, the microenvironment for a tumor is hypoxic and HIF1-α is upregulated, thereby causing the upregulation of VEGF and inducing tumor angiogenesis. Therefore, it is necessary to perform anti-angiogenesis therapy under such a condition. Presently, sorafenib and lenvatinib are recommended as the principal multi-target inhibitors for HCC systemic therapy. In the TACTICS and TACTICS-L study, the combination therapy of TACE and MTDs has been demonstrated to significantly prolong PFS and OS of HCC patients at the early or middle stage (14). These results suggest that the combination of MDTs and TACE has a great therapeutic value and application prospect to partially unresectable HCC patients. However, these studies exclude advanced HCC cases. For advanced HCC, most patients face the problem of bulky masses, tumor vascular invasion, and low KPS score, and most of them fail to have their masses completely embolized after TACE therapy, resulting in poor efficacy. In 2019, the combination therapy of pembrolizumab with lenvatinib was approved by FDA as the first-line therapy for advanced HCC. Its efficacy in the disease control rate was almost not inferior to that of tecentriq combined with bevacizumab in the Imbrave150 study (15). These results completely illustrate the inevitable trend of combining immunotherapy with targeted anti-angiogenesis.

In the present study, when compared with the PD-1 group, the duplex group achieved a median PFS of 4.73 months more, HR 0.44, significantly reduced risk of disease progression, and acceptable toxic and side effects. In terms of the complete response and disease control rate, the duplex group showed better results when compared with the PD-1 group, thus indirectly illustrating the significant potential of tumor regression. On one hand, MTDs exerted an anti-tumor effect by themselves, targeting multiple proteins that promoted tumor growth. On the other hand, after conventional TACE, tumors lacked oxygen and the activity of immune cells infiltrating tumor tissues could be inhibited in an acidic microenvironment. Meanwhile, anticancer drugs and cytolytic T-lymphocyte were difficult to deliver to the tumor site owing to disordered blood vessels inside the tumor. In this case, multi-target inhibitors induced the normalization of tumor vascular and reshaped the tumor microenvironment while inhibiting tumor angiogenesis, thus further improving the efficacy of combined therapy. Therefore, the combination of sequential immunotherapy with multi-target inhibitors after performing TACE can not only increase the anti-tumor effect but also improve the microenvironment for tumor tissues. They are both mutually complementary, which improves the overall therapeutic effectiveness. In terms of the median OS, the duplex group was numerically superior to the PD-1 monotherapy group, albeit the difference was not statistically significant. With the extension of time, the OS rate at the 18th month was higher than that of the PD-1 group, and the difference was statistically significant. This event may have been related to the small sample size and the influence of subsequent treatment on OS. In addition, the PFS was helpful for the OS. However, whether the prolongation of the OS in the duplex group resulted from the prolongation of the PFS remains to be explored.

Tumor size, optimal efficacy, and combination therapy with MTDs are the factors that affect the PFS of patients with advanced HCC. Cox multivariate regression analysis revealed that the combination with sorafenib and achievement of CR/PR after TACE acted as independent prognostic factors affecting PFS of HCC patients who received sequential immunotherapy after TACE. This event may be attributed to the fact that some patients cannot achieve the effect of inactivating macro lesions by TACE owing to their excessive large tumor sizes, and they even fail to achieve CR/PR after multiple TACE operations. The remaining lesion is an important cause of liver cancer recurrence, further inducing neovascularization after TACE, which leads to the recurrence in a short time. TACE (≤2) frequency, optimal efficacy, tumor size, CNLC stage-IIIa, and the combination therapy with MTDs are the factors affecting OS of advanced HCC. Meanwhile, CNLC stage-IIIa, TACE frequency ≤2, and the achievement of CR or PR acted as independent influencing factors of OS. Indeed, the present study is a retrospective analysis with limited evidence. Therefore, some subjective factors and statistical sample bias were introduced in the study owing to limitations such as inconsistent times of TACE and immunotherapy, inconsistent immunotherapy drugs, small sample size, and inconsistent multi-target inhibitors. In terms of safety, the common adverse events included hematological toxicity and liver dysfunction, most of which were of grade 1–2. Thyroid dysfunction was noted to be a common immune-specific adverse reaction, albeit no serious immune-related adverse reactions were recorded.

In conclusion, TACE can enable rapid elimination of tumor tissues and promote the generation of a large amount of tumor neoantigen for anti-tumor immune response, thus activating immunity. By combined therapy of TACE and PD-1, the immune cells can be relieved from suppression, thus stably contributing to the anticancer effect. On this basis, the combination of sorafenib may enable further improvement of patients’ PFS and thus prolong their OS.

## Data Availability Statement

The raw data supporting the conclusions of this article will be made available by the authors, without undue reservation.

## Ethics Statement

The studies involving human participants were reviewed and approved by the Second Affiliated Hospital of Chongqing Medical University. Written informed consent for participation was not required for this study in accordance with the national legislation and the institutional requirements.

## Author Contributions

JQ and SY conducted the experiments. SY and HZ collected the data. JQ and SY conducted the data analysis. JQ and YH wrote the manuscript. All authors listed have made a substantial, direct, and intellectual contribution to the work and approved it for publication.

## Funding

The study was supported by the Chongqing Medical Scientific Research Project (No. 2021MSXM086).

## Conflict of Interest

The authors declare that the research was conducted in the absence of any commercial or financial relationships that could be construed as a potential conflict of interests.

## Publisher’s Note

All claims expressed in this article are solely those of the authors and do not necessarily represent those of their affiliated organizations, or those of the publisher, the editors and the reviewers. Any product that may be evaluated in this article, or claim that may be made by its manufacturer, is not guaranteed or endorsed by the publisher.
